# Reproductive Health in Congenital Heart Disease: Preconception, Pregnancy, and Postpartum

**DOI:** 10.3390/jcdd10050186

**Published:** 2023-04-22

**Authors:** Dan G. Halpern, Christina A. Penfield, Jodi L. Feinberg, Adam J. Small

**Affiliations:** 1 NYU Adult Congenital Heart Disease Program, Leon H. Charney Division of Cardiology, NYU Grossman School of Medicine and NYU Langone Health, New York, NY 10016, USA; jodi.feinberg@nyulangone.org (J.L.F.); adam.small@nyulangone.org (A.J.S.); 2 Division of Maternal-Fetal Medicine, Department of Obstetrics and Gynecology, NYU Grossman School of Medicine and NYU Langone Health, New York, NY 10016, USA; christina.penfield@nyulangone.org

**Keywords:** congenital heart disease, pregnancy, cardio-obstetrics, contraception

## Abstract

The prevalence of congenital heart disease (CHD) in pregnancy is rising due to the improved survival of patients with CHD into childbearing age. The profound physiological changes that occur during pregnancy may worsen or unmask CHD, affecting both mother and fetus. Successful management of CHD during pregnancy requires knowledge of both the physiological changes of pregnancy and the potential complications of congenital heart lesions. Care of the CHD patient should be based on a multidisciplinary team approach beginning with preconception counseling and continuing into conception, pregnancy, and postpartum periods. This review summarizes the published data, available guidelines and recommendations for the care of CHD during pregnancy.

## 1. Introduction

Congenital heart disease (CHD) is the most common type of heart disease in pregnancy in the developed world, and cardiovascular disease (CVD) is the leading cause of non-obstetric pregnancy-related mortality in the United States [1,2,3]. Due to advances in pediatric cardiology care and congenital cardiac surgery, there are now more adults than children living with CHD, with 90% of children diagnosed with CHD surviving into adulthood [4]. However, CHD in pregnancy carries significant risk of both maternal and fetal morbidity [2]. CHD in pregnancy carries significant risk of complications and increased length of stay during delivery hospitalization, as well as risk of readmission after discharge. Maternal complications may include heart failure, arrhythmia, thromboembolic disease, endocarditis, pre-eclampsia, or hemorrhage [5,6]. Fetal risks include premature birth, intra-uterine growth restriction (IUGR), small-for-gestational-age (SGA), CHD and neonatal death. In addition, acquired CVD risks such as obesity, hypertension and diabetes as well as advanced maternal age may further complicate pregnancy [1,2].

Care for the adult congenital heart disease (ACHD) patient begins with a thoughtful pre-conception counseling and risk stratification with an adult congenital cardiologist and maternal-fetal-medicine (MFM) specialist. In the majority of cases, pregnancy is well tolerated by the CHD patient, yet anticipation of specific complications is key. A multidisciplinary cardio-obstetric team approach including obstetricians, congenital cardiologists, maternal fetal medicine, geneticists, anesthesiologist, pharmacists, clinical psychologists, and nursing in an experienced healthcare center provides the infrastructure for a safe pregnancy [7].

This review summarizes the guidelines and recommendations for pregnancy and CHD, including pre-conception counseling, pregnancy, and labor and delivery.

## 2. Physiological Changes during Pregnancy and Labor

**Figure 1:**
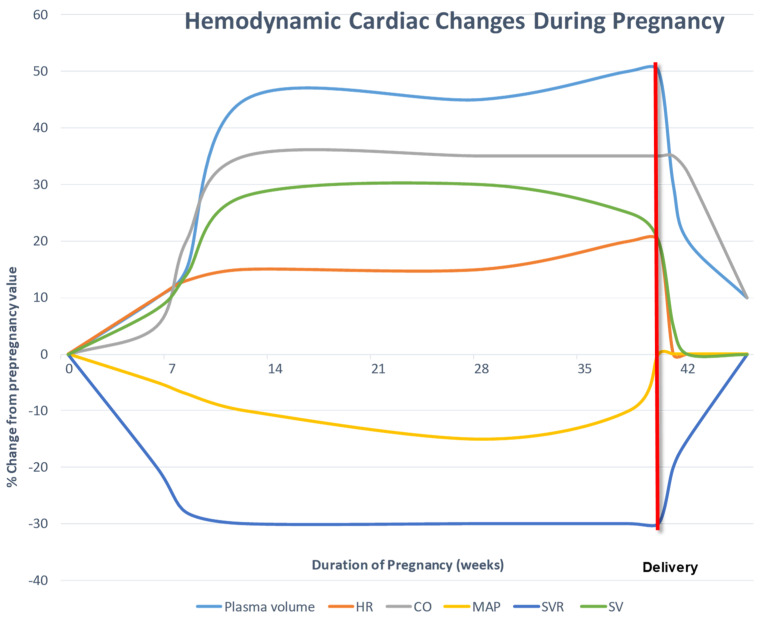
Hemodynamic Cardiac Changes during Pregnancy, Peripartum and Postpartum by week. CO = cardiac output; HR = heart rate; MAP = mean arterial pressure; SV = stroke volume; SVR = systemic vascular resistance.

The physiological changes of pregnancy are profound and may uncover latent hemodynamically significant cardiac disease [8] (Figure 1). Major hemodynamic changes include increase in plasma volume (↑ 40%), cardiac output (↑ 30–50%), heart rate (↑ 10–15 beats per minute) with a decrease in systemic and pulmonary vascular resistance [9]. There is relative anemia due to a lesser increase in red blood count compared to plasma volume. During delivery, cardiac output increases up to 80% immediately postpartum and there is a large volume auto-transfusion of blood from the utero-placental circulation with increase in pre-load [10]. The systemic and pulmonary vascular resistance rapidly rises within the first 24–72 h postpartum and is considered the vulnerable period for hemodynamic decompensation. Most hemodynamic changes resolve within 2 weeks postpartum, yet return to complete pre-pregnancy state may take several months. Pregnancy is also considered a hypercoagulable state peaking at the early post-partum period and is considered to extend into the fourth trimester (first 6 weeks postpartum).

## 3. Preconception Counseling and Risk Scores

ACHD patients considering pregnancy should receive timely preconception counseling because the physiologic changes in the cardiovascular system during pregnancy may confer risk for those who are not able to appropriately adapt. Ideally, this counseling should occur in a setting which includes a multi-disciplinary cardio-obstetrics team who has experience of CHD. During the visit, the provider can discuss not only the potential medical risks and considerations in the pregnancy dyad, but also address the larger sociocultural and ethical considerations that may be involved in the decision for pregnancy, particularly in high-risk patients where the condition is not compatible with pregnancy. Neonatal and maternal risks are intertwined and maternal risk factors such as decreased functional capacity, cyanosis, smoking, use of anticoagulation and mechanical valves have all been associated with poorer neonatal outcomes. Important conditions in which pregnancy is not recommended include Eisenmenger syndrome and pulmonary hypertension, cyanosis, Fontan circulation with complications, certain high-risk aortopathies, severe systemic ventricular function, severe aortic coarctation, severe mitral stenosis and symptomatic aortic stenosis [2].

The preconception consult will ultimately center on the cardio-obstetric team’s ability to predict the patient’s maternal cardiovascular risk during the pregnancy. This is accomplished by assessing the patient’s condition, including medical history, functional class, oxygen saturation levels, natriuretic peptide (BNP) levels, cardio-imaging of anatomy and ventricular function, congenital lesion, intrapulmonary pressures and aortic diameters, exercise capacity, and arrhythmias. It is accepted that pregnancy is favorable when exercise capacity exceeds 80% [2]. Cardiovascular medications should be adjusted as explained below.

Several risk score models have been developed to assist providers in synthesizing this information for the purpose of risk stratification, each one with relative advantages and disadvantages. These include the modified world health organization (mWHO) [2,11] risk classification, CARdiac disease in PREGnancy II (CARPREG II) [12], and ZAHARA [13] (Table 1).

A typical approach is to first estimate disease-specific risk using the modified World Health Organization (mWHO) risk classification. The mWHO risk classification provides an important initial step in the recognition of risks, including the ability to quickly determine whether referral to a center with tertiary or quaternary care would be recommended during pregnancy. The mWHO classification is divided into four categories. mWHO Class I consists of mild congenital heart disease that is not associated with significant risk of morbidity and mortality compared to the general pregnant population. The risk of pregnancy gradually increases to extremely high risk in mWHO class IV, where the pregnancy may pose such a significant health risk such that it is contraindicated. 

While the mWHO provides an essential first impression based on heart lesion, often there is considerable variation amongst this class of patients and further refinement using the other risk stratification tools is necessary. Therefore, additional risk prediction models using clinical factors have been developed. The initial Cardiac Disease in Pregnancy (CARPREG) multicenter study was the first to pioneer prediction models for cardiovascular disease in pregnancy over 20 years ago [14]. Since then, it has been updated with additional data from almost 2000 high-risk pregnancies to the CARPREG II risk score, which is now the most contemporary risk prediction model. CARPREG II incorporates now the patient’s history (prior cardiac events, NYHA class), physical exam (O2 sat < 90%), specific lesions (e.g., mechanical valves, coronary artery disease, high-risk aortopathy), imaging (systemic ventricular dysfunction, left-sided obstruction, pulmonary hypertension) and presence of a late first antenatal visit. The range 0–1 points pertains to up to 5% cardiovascular risk in pregnancy and >4 points predicts a high-risk pregnancy with 40% risk and above. It should be noted that CARPREG II does not distinguish between systemic ventricular functional severity and includes Marfan syndrome and bicuspid valve disease both as high-risk aortopathies, although they differ in aortic dissection prevalence. Prior studies have demonstrated that CARPREG II predictors can provide additive information when used in conjunction with the mWHO risk classification system. The multinational Registry On Pregnancy and Cardiac disease (ROPAC) registry summarized outcomes from 1321 pregnancies of women with CVD from 28 countries between 2007 and 2011. They showed improved accuracy of the mWHO classification, by adding pre-pregnancy atrial fibrillation and signs of heart failure to the classification [15].

The ZAHARA study proposed an additional risk scoring system for predicting pregnancy complications exclusively for pregnant patients with CHD. Most notably, the investigators proposed that the addition of two factors not included in CARPREG II (cardiac medication use and atrioventricular valve regurgitation) would enhance prediction performance. However, validation studies comparing the three available prediction tools (mWHO, ZAHARA, and CARPREG II) in different cohorts with preexisting cardiac lesions have had variable results, likely related to the clinical characteristics of the cohort (congenital versus acquired cardiac lesions), the location and services available where care was received, and patient factors (e.g., late presentation to care, access to preconception optimization). Overall, these models are helpful for initial stratification and counseling, yet each case requires a further in-depth lesion-specific evaluation and a tailored risk appraisal.

Regardless of the identification of a specific genetic mutation, first-degree family members have a higher risk of CHD. As a result, preconception counseling should also include referral to genetic counseling. During pregnancy, fetal echocardiogram should be advised at around 20–22 weeks gestation in addition to a detailed anatomy scan [2,4,16].

## 4. Contraception

Contraception should be routinely discussed with all ACHD patients of childbearing age, with the goal of reviewing safe and appropriate options for these unique patients (Table 2). In a cross-sectional survey of ACHD patients, more than half of women had not received contraceptive or reproductive counseling related to their cardiac condition [17]. Another study of 100 women with CHD of child-bearing age demonstrated that 45.4% of 141 pregnancies that occurred were unexpected [18]. In order to provide safe contraceptive guidance, clinicians should understand the impact of contraceptive hormones, such as estrogen and progesterone, on patients with CHD. Current contraceptive options involve combination estrogen with progesterone (most common pill formulations, vaginal ring, and patch), progesterone only (intrauterine device (IUD) and specific pill combinations), and hormone-free methods (barrier methods such as condoms, copper IUD, and sterilization). Estrogen increases the risk of venous thromboembolism and hypertension, while progesterone is associated with fluid retention and weight gain. Hormone-free methods may have other unpleasant side effects or decreased efficacy, making the decision for contraception in patients with CHD very complex [19].

Both 2018 AHA/ACC and the 2020 ESC ACHD guidelines advise avoiding estrogen-containing birth control in those with CHD and the following conditions: prior thrombotic events, cyanosis, Fontan physiology, pulmonary arterial hypertension and mechanical valves [4,16]. The ACOG practice bulletin recommends avoiding estrogen-containing contraception for the following additional scenarios: smoking and age 35 years or older, less than 21 days after giving birth, 21–42 days after giving birth with peri-partum cardiomyopathy, history of deep venous thrombosis (DVT) or pulmonary embolism (PE), hereditary thrombophilia, superficial venous thrombosis, and diabetes > 20 years or diabetes with microvascular disease [19]. Lastly, the 2016 US Medical Eligibility Criteria (MEC) for Contraceptive Use provides a broader list of groups that should avoid estrogen-containing contraceptives [19]. Providers must consider that their patients with Fontan circulation or right heart disease are at increased risk for congestive hepatopathy, liver fibrosis and progression to liver cirrhosis that would further impact decisions regarding contraception. 

Though progesterone-containing contraception is generally considered lower risk for those with CVD, due to a lower risk of thrombosis, it should be avoided in some high-risk conditions. The US MEC for Contraceptive Use states that the depot medroxyprogesterone acetate is not recommended for patients with multiple risk factors for atherosclerotic CVD, vascular and ischemic heart and brain disease, as well as liver cirrhosis [19].

Intrauterine device (IUD) is a valuable option for contraception, especially when there is intolerance, contraindication or inconvenience using oral or transdermal hormonal therapy. IUDs are extremely effective at preventing pregnancy while providing a safe contraception method in high-risk patients with CHD. There are currently two classes of IUDs: copper (non-hormonal) and progesterone-containing (hormonal). The copper-based IUD does not interfere with menstrual cycle timing but has an increased risk of bleeding during menses, and therefore may not be the preferred option for patients taking anticoagulation or with significant anemia. The progesterone-based IUD is exceptionally effective (>99%), and decreases bleeding during menstruation (even often inducing amenorrhea). Rarely, it is associated with weight gain and minimal edema.

There are considerations for the procedures associated with contraception. IUD insertion is generally considered a low-risk procedure; yet in high-risk ACHD patients such as Fontan circulation, cyanosis, Eisenmenger syndrome and pulmonary hypertension, where a vasovagal reaction may cause a hemodynamic compromise, these procedures should be performed in a hospital setting under close observation and ACHD expertise. The risk of endocarditis after IUD insertion is likely low and subacute bacterial endocarditis prophylaxis administration is at the clinician’s discretion. In the case of tubal ligation, if a laparoscopic approach is used, abdominal insufflation may reduce venous return, which may produce deleterious effects in a Fontan circulation. For these patients, laparotomy is advised.

There are interactions between contraception and common cardiac medications. Bosentan decreases the levels of ethinyl estradiol and norethindrone, which may decrease the efficacy of combined oral contraceptives. Because depot medroxyprogesterone acetate and warfarin have potential long-term consequences on bone density, depot medroxyprogesterone acetate should be individualized for use in patients who were treated with warfarin as children. Patients on hormonal birth control and warfarin require frequent INR checks, as hormonal contraceptives may change the serum concentration of vitamin K antagonists. Hormonal contraceptives can also adversely affect lipid profile, which should be considered when utilized in patients with atherosclerotic risk factors. Lastly, contraception options that reduce menstruation, such a progesterone-containing IUDs, may be beneficial in patients on anticoagulation or patients with anemia that exacerbates their CHD.

## 5. Cardiovascular Medications Use during Pregnancy

The use of cardiovascular drugs during pregnancy requires careful pre-conceptual planning recognizing that drugs affect both mother and fetus [21]. The FDA pregnancy and lactation labeling rule (PLLR) is now replacing the longtime used FDA ABCDX designation system and the latter is no longer recommended for clinical use due to its inaccuracies in risk stratification. Contrary to popular notion, only a minority of medications are teratogenic in pregnancy. However, due to medico-legal concerns, testing and evaluating safety of medications during pregnancy is challenging and research is limited.

Pharmacokinetic changes during pregnancy include increase in liver and kidney clearance, changes in liver enzymatic activity, decrease in gut absorption, increase in gastric pH, plasma dilution with decrease in plasma binding proteins, and increase in volume of distribution which commonly result in a decrease in net effect of a drug. The most commonly used CVD medications used in pregnancy are furosemide and beta-blockers. Several drugs are contraindicated in pregnancy including angiotensin converting enzyme inhibitors (ACEi), angiotensin receptor blockers (ARBs), angiotensin receptor neprilysin inhibitor (ARNi), aldosterone antagonists, endothelin receptor antagonists (e.g., bosentan, macitentan), riociguat, statins, atenolol and amiodarone. Beta-blockers such as metoprolol and propranolol are considered safe and their adverse effects such as small for gestation age fetus, neonatal bradycardia and hypoglycemia are more prevalent at an equivalent metoprolol dose of 50 mg and above [22]. Diuretics are used for hypervolemia and heart failure, yet may cause electrolyte imbalances and decrease in utero-placental perfusion. Anticoagulants such as heparin and enoxaparin do not cross the placenta and increase the incidence of maternal bleeding. Warfarin, by contrast, crosses the placenta and increases incidence of fetal bleeding. The risk of warfarin related embryopathy is decreased with a daily dose equal to or less than 5 mg during the first trimester. Yet, in the latter trimesters warfarin can be used in higher dosages. 

It should be emphasized that during pregnancy, life-saving medications should not be withheld during emergency situations. 

## 6. Congenital Valve Disease

Valvular heart disease constitutes the majority of heart disease in pregnancy. CHD and rheumatic heart disease are major causes [23]. By and large, regurgitation is better tolerated than stenosis because valvular regurgitation allows for an increase in forward stroke volume during stress states as well as the low vascular resistance state. See Table 3 for a content summary.

In congenital aortic or mitral valve stenosis, it is observed that transvalvular gradients, as assessed by Doppler echocardiography, will increase between the first and second trimester. This phenomenon is likely flow-mediated as calculated effective valvular orifice area will remain stable [31]. Care should be taken in the interpretation of valve stenosis severity in pregnancy.

Severe mitral stenosis, which may occur in isolation or in association with other lesions (as in Shone Complex, for example, a syndrome of serial left-sided obstructions), can be considered the most dangerous valve lesion in pregnancy. According to a meta-analysis, mortality in pregnancy is estimated at 3%. Major morbidity is also common, with 37% experiencing pulmonary edema and 16% arrhythmia [24]. Fetal outcomes may also be compromised; pregnancy loss and preterm delivery have been observed in 6% and 18% of pregnancies, respectively [24]. 

Aortic valve stenosis may occur in isolation or, like mitral stenosis, in association with other left-sided obstructions or other lesions such as septal defects. Mortality from severe aortic stenosis in pregnancy is approximately 2%, with 9% incidence of pulmonary edema and 4% incidence of arrhythmia [24]. Morbidity correlates with lesion severity and symptomatology. Patients with symptomatic severe aortic valve stenosis have been observed to experience a much higher rate of hospitalization for cardiac indications during pregnancy than those without symptoms or severe disease [25]. Even after pregnancy, the clinical effects of pregnancy may linger for patients with moderate or severe aortic stenosis. Compared to those not experiencing pregnancy, the rate of valve intervention > 1 year postpartum may be higher. Finally, as with mitral stenosis, fetal outcomes are affected. Severe aortic stenosis is associated with low birth weight, as well as a 5% rate of pregnancy loss and 4% rate of preterm delivery [24,25].

Pulmonary stenosis is thought to be well tolerated in pregnancy, based on limited data. In a case–control study matching 17 patients with pulmonary stenosis (seven severe) with a matched cohort. While two patients had worsening functional status in pregnancy, no serious cardiac events were observed [26].

Valvular regurgitation in pregnancy carries lower complication rates than those observed with valvular stenosis. Of 430 pregnancies with valvular regurgitation (73% with CHD) studied by Pfaller and colleagues, 5 (1%) resulted in either cardiac death or resuscitated arrest. Major morbidity, most commonly heart failure, was observed in 13% of pregnancies overall. Semilunar valve regurgitation was better tolerated than atrioventricular valve regurgitation. Mitral or tricuspid regurgitation carried morbidity rates three to five times higher than aortic or pulmonary valve regurgitation. Pulmonary hypertension, left ventricular systolic dysfunction, and prior cardiac events all predicted adverse outcomes. Fetal and neonatal death occurred in 2%, a lower rate than that observed in mitral or aortic stenosis in other studies. Still, major adverse fetal events, most commonly small for gestational age and preterm delivery, were observed in 31% of pregnancies with valvular regurgitation [27].

Bioprosthetic valves are commonly used in the palliation of CHD. Not surprisingly, structural valve degeneration portends higher risk of maternal cardiac complications in pregnancy. In a series by Wichert-Schmitt and colleagues, 27% of prosthetic valves encountered had significant stenosis or regurgitation. Higher maternal age and left-sided prosthesis (compared to right) predicted higher risk of maternal adverse events [28]. The possibility of pregnancy accelerating bioprosthetic valve degeneration has been raised, but data on this topic remain equivocal [32,33]. 

Mechanical prosthetic valves may carry a similar risk of maternal mortality to bioprosthetic valves according to a large prospective registry [29], but chronic anticoagulation raises difficult questions and causes significant morbidity. The risk of a serious adverse maternal event in pregnancy is approximately 42% for a patient with a mechanical valve, compared to 21% with a bioprosthetic valve. In pregnant persons with mechanical valves, adverse events may include valve thrombosis (with or without systemic embolism) or hemorrhage [29,34]. Generally speaking, vitamin K antagonists have been associated with better maternal outcomes, whereas heparin products have been associated with better fetal outcomes. In a large meta-analysis, vitamin K antagonists carried a 5% risk of death, systemic embolism, or heart failure—one third the observed rate of complications on heparin [34]. However, vitamin K antagonists have been associated with a three-fold increased risk of miscarriage in the first trimester, and more than double the rate of pregnancy loss or congenital defects compared with heparin [29,34]. As previously mentioned, when the dose of warfarin is 5 mg or less, fetal risks of vitamin K antagonists are no worse than those observed with heparin [34].

In rare circumstances, urgent invasive intervention is warranted during pregnancy. Sometimes this circumstance is foreseeable, or pregnancy occurred with inadequate counseling. Other times, emergencies may manifest unexpectedly, as in cases of endocarditis. Additionally, a previously borderline lesion may be poorly tolerated given the significant physiologic changes occurring in pregnancy.

Maternal mortality from cardiac surgery in pregnancy has been reported as 7%, and may not differ depending on the trimester during which surgery is performed. Neonatal mortality on cardiopulmonary bypass has been reported between 16 and 33% due to placental hypoperfusion [35,36]. When possible (the decision typically depending on gestational age), cesarean delivery prior to cardiac surgery may improve fetal outcomes [37,38].

Transcatheter valve procedures have been performed in pregnant persons requiring intervention. Balloon mitral valvuloplasty may not be possible in patients with CHD, but aortic valvuloplasty is commonly undertaken in this population. Favorable maternal outcomes have been observed with balloon valvuloplasty [23]. In the current era, transcatheter valve replacement can be performed. Transcatheter valve replacement during pregnancy has been reported in all four valve positions, in carefully selected patients [39,40,41,42]. Besides refractory heart failure and valve thrombosis, endocarditis may warrant surgical or interventional consideration in pregnancy. Mortality of endocarditis in pregnancy is estimated to be approximately 11%. However, a systematic review observed that 80% of pregnancies complicated by endocarditis resulted in delivery and survival to discharge, perhaps suggesting a bi-modal distribution of outcomes [30]. Data on endocarditis prophylaxis are limited, but antibiotics before anticipated delivery are still recommended in selected patients such as those with prosthetic heart valves or history of endocarditis [2].

## 7. Specific Congenital Heart Disease Lesions

See Table 4 for content summary.

### 7.1. Shunt Lesions

Atrial septal defect (ASD) constitutes the most common adult CHD lesion. It causes left-to-right shunting with right-sided chamber enlargement, which in pregnancy may result in right ventricular dysfunction, arrhythmia, development of endocarditis and in rare circumstances, propagate a paradoxical embolus. Anomalous pulmonary venous connection (APVC) is another lesion overloading the right heart. Commonly, a single APVC without an ASD (i.e., sinus venosus defect) does not cause significant right-sided volume increase and is not associated with paradoxical emboli. The development of pulmonary hypertension in ASDs or APVCs is infrequent, yet may be more severe when it develops due to early right ventricular involvement. Patent Foramen Ovale (PFO), a residual flap communication between the atria, may become a cause for symptomatic right-to-left shunting resulting in desaturation or paradoxical emboli. Commonly, unrepaired ASDs are well tolerated during pregnancy, yet embolic complications have been reported in 5% of pregnant ASD patients so meticulous intravenous care, use of micron-filter intravenous lines and DVT prophylaxis is recommended. Full anticoagulation is reserved for high-risk patients, such as those with atrial arrhythmia or thrombophilia and may be considered in those with indwelling intra-cardiac devices such as pacemaker leads [2,43]. In an older study, unrepaired ASDs have been associated with increased risk of pre-eclampsia, SGA and increased fetal death [50]. Device closure of a symptomatic right-to-left shunting is rarely required, but is feasible with appropriate fetal protection from radiation and preferably after the first trimester, when organogenesis is complete. The risk of complications of repaired ASDs in pregnancy depends on the integrity of the closure device or surgical patch, function of the right ventricle (RV) and degree of pulmonary hypertension.

Ventricular septal defect (VSD) and patent ductus arteriosus (PDA) are lesions that result in left-sided chamber enlargement and are more prone to the development of pulmonary hypertension if left unrepaired. However, small VSDs and PDAs without pulmonary hypertension are not expected to have any hemodynamic effect on pregnancy. Unrepaired VSDs have also been implicated in an increased risk of pre-eclampsia [51]. On the contrary, patients with large shunts associated with pulmonary hypertension are advised against pregnancy [2].

### 7.2. Tetralogy of Fallot

Repaired tetralogy of Fallot (TOF) constitutes the most common moderate complexity lesion in adults and is categorized as mWHO risk class II [2]. Its pregnancy-related complications (which occur in 8%) include arrhythmia (mainly atrial arrhythmia), worsening pulmonary and tricuspid regurgitation, right ventricular dysfunction (both systolic and diastolic), endocarditis and thromboembolism [43]. In one study, the most important predictor of complications of repaired TOF was use of cardiac medications prior to pregnancy [52]. In patients with mild to moderate right ventricular dilatation, pregnancy is unlikely to worsen size or function of the ventricle [53]. However, it is reasonable to discuss the possible need for pulmonary valve intervention prior to pregnancy, especially with larger sized RV or with signs of dysfunction. Unrepaired TOF (mWHO risk class III) patients are cyanotic and may have been palliated with a systemic-to-pulmonary shunt to ensure pulmonary blood flow. These patients carry a high risk of paradoxical thromboembolic events as well as cyanotic complications and are counseled against pregnancy. Maternal screening for 21q11 deletion is recommended during pre-conception counseling for all TOF patients [4,16].

### 7.3. Coarctation of the Aorta

Coarctation of the aorta (CoA) is associated with hypertension and aortic dissections. Turner syndrome patients are in particular high risk of aortic dissections, especially in the presence of a dilated aorta and bicuspid aortic valve. Severe CoA is categorized as mWHO risk class IV and patients are recommended to undergo CoA intervention prior to the pregnancy or avoid pregnancy [2]. Complications are related to the fixed afterload causing left ventricular failure, decreased forward flow through the obstruction to the placenta, inherent aortopathy, and the increased prevalence of brain aneurysms. Smaller aortic dimensions (<1.2 cm) are associated with more hypertensive events [54]. However, there are emerging data suggesting pregnancy outcomes are better than previously suggested. In an ROPAC prospective observation study, 303 pregnant women with CoA had a maternal event rate of 4.3% (heart failure 3.3%, arrhythmia 1%) and hypertensive disorders occurred in 6.3%, not exceeding the average of the general population [55]. In the event of refractory hypertension, an aortic stent may be delivered during pregnancy with appropriate fetal protection.

### 7.4. Ebstein Anomaly

Ebstein anomaly includes apically positioned and abnormal tricuspid valve, right ventricular myopathy, Wolff–Parkinson–White (WPW) syndrome and frequently atrial communications. Arrhythmia has been reported in 3.9% and heart failure in 3.1% [43]. Atrial arrhythmias, especially atrial fibrillation in the setting of WPW, may degenerate to a life-threatening ventricular arrhythmia. Tolerance of pregnancy is related to the degree of tricuspid regurgitation and right ventricular function as well as the burden arrhythmia and presence of cyanosis. It is recommended not to proceed with pregnancy in Ebstein anomaly in the presence heart failure or hypoxemia (with <85% saturations) [2]. As with other complex lesions, fetal and neonatal complications may occur, including prematurity, small size for gestational age, or less commonly, death. In a review of 128 pregnancies in mothers with Ebstein anomaly, fetal death was not observed but perinatal death occurred in three cases [43].

### 7.5. Transposition of the Great Arteries

d-transposition of the great arteries (TGA) nowadays undergo an arterial switch operation (ASO) which restores normal ventriculo–arterial connections. The adult complications of the ASO include coronary compression, neo-aortic dilatation and regurgitation as well as supra-pulmonary valve stenosis. Pregnancy is well tolerated with ASO with a low rate of complications such as heart failure, which may occur in the setting of neo-aortic regurgitation and arrhythmia [44]. However, d-TGA post atrial switch operation (i.e., Senning or Mustard) and congenital corrected TGA (cc-TGA or L-TGA) both have a systemic RV and are at increased risk for developing heart failure or arrhythmia. Complications of d-TGA following atrial switch include worsening in systemic ventricular failure and worsening in systemic tricuspid regurgitation, arrhythmia, prematurity, and SGA. Worsening of systemic ventricular function or tricuspid regurgitation may not be reversible [45,46]. Canobbio et al. reported the outcome of 70 pregnancies of women with atrial switch. In total, 36% developed cardiac complications out of which 9% (one-quarter of complications) were heart failure, primarily in the second and third trimester—one patient required heart transplantation and another died suddenly one month after delivery [46]. For systemic RVs, there is a Class IIa recommendation against pregnancy by the ESC when NYHA class III or IV, systemic ventricular dysfunction (EF < 40%) or severe tricuspid regurgitation is present. Frequent evaluation for arrhythmia and surveillance of the systemic right ventricular function is recommended during pregnancy [2]. Patients with cc-TGA commonly have a dysplastic or Ebstenoid-appearing tricuspid valve that may drive systemic RV dysfunction and may need to be intervened upon prior to pregnancy.

### 7.6. Cyanosis

Cyanotic conditions (unrepaired or palliated) are associated with a significant risk of both maternal and fetal complications. As systemic vascular resistance decreases during pregnancy, right-to-left shunting increases, worsening cyanosis and increasing the risk of paradoxical emboli. Maternal oxygen saturation ≤ 85% portends increased risk of complications and a very low live birth rate of 12% [43]. The risk of thrombosis is further increased due to the hyperviscosity caused by erythrocytosis. In a meta-analysis, heart failure was reported in 18.9%, arrhythmia in 4.8% and endocarditis 4.1% [43]. Without Eisenmenger syndrome (ES), live births are noted to be 43% with high burden of prematurity. Care includes filtered vascular lines, compression stockings, sequential compression devices, treatment of iron deficiency and thromboembolic prophylaxis.

ES complicating cyanosis is associated with high rates of heart failure (21%) and thromboembolic complications (18.8%). Previous maternal mortality was reported to be between 20 and 50% and nowadays has improved to reports ranging from 10.3 to 23% [56,57]. However, it continues to carry a prohibitively high risk in pregnancy. Only one-quarter of pregnancies with ES proceed to term with a majority of fetuses born prematurely and fetal mortality reaching 30%. Patients with ES are recommended to undergo a permanent form of sterilization.

Treatment of ES during pregnancy includes in addition to the aforementioned shunt care advanced pulmonary hypertension therapies such as phosphodiesterase-5 inhibitors (e.g., sildenafil) and prostaglandins (e.g., treprostinil). As eluded to before, pulmonary vascular resistance increases 24–72 h after delivery in hemodynamic decompensation may develop and therefore admission to the intensive care unit for post-partum observation is recommended [45].

### 7.7. Single Ventricle with Fontan Palliation 

Once any complication occurs with the Fontan circulation, pregnancy is contraindicated, categorized as mWHO risk class IV [2]. Younger patients with good single ventricular function, low systemic venous Fontan pressure and no significant collateral circulation or liver disease can be categorized as mWHO risk class III and undergo a high-risk pregnancy. Fontan palliated patients are at increased risk of arrhythmia (3–37%, mostly atrial) and ventricular failure (3–11%) [43,47]. Miscarriage is common (30–45%) and there is an increased risk of prematurity (28–59%), peripartum bleeding, SGA, and neonatal death [48,58]. The ESC guidelines recommend against pregnancy in a Fontan circulation when saturations are <85%, or when there is depressed single ventricular function, moderate to severe atrioventricular valve regurgitation, refractory arrhythmia, or protein losing enteropathy. Anticoagulation is recommended to all Fontan palliated patients undergoing pregnancy [2]. 

## 8. Delivery Planning

While CHD carries an increased risk of both maternal and fetal complications, the absolute rates of these complications (and of mortality) are low during admission for delivery, occurring in less than 0.5% [6]. While early term delivery has not demonstrated benefit at reducing maternal morbidity in CHD, term delivery has actually been shown to reduce risk of complications for pregnant persons with CHD [2,59,60]. Due, presumably, to heightened concern for pregnant CHD patients, pregnant persons with CHD are more likely to undergo cesarean delivery than those without CHD. However, registry data do not suggest that planned cesarean delivery improves maternal outcomes [61]. Success has been observed with a strategy of planned vaginal birth in the majority of cases, including Valsalva, unless obstetric indications warrant cesarean delivery. The authors use assisted second stage in cases of asymptomatic severe aortic stenosis or lower-risk aortopathy [62]. According to ESC guidelines, cesarean delivery is recommended in cases of oral anticoagulant use, significant aortopathy, intractable heart failure, or severe pulmonary hypertension [2].

Medications used for induction of labor may carry adverse cardiac effects. Misoprostol and dinoprostone can cause vasodilation but are generally well tolerated. When systemic vasodilation must be avoided (e.g., severe aortic or subaortic stenosis), a cervical ripening balloon has been recommended [2]. Artificial rupture of membranes is considered safe; and while oxytocin may cause vasodilation and even ECG changes, especially when administered as an intravenous bolus, this complication is rarely observed [2,63].

Patients on systemic anticoagulation warrant special attention around the time of delivery, particularly if neuraxial analgesia is used. It is recommended that unfractionated heparin be reversed with protamine sulfate. For patients on low molecular weight heparin, protamine is recommended, but reversal is incomplete so some bleeding may persist. Prothrombin complex concentrate is the preferred method to reverse INR in patients on vitamin K antagonists. Vitamin K itself may be used but its effect may not be observed for 8–12 h [2].

The manner of cardiac monitoring in labor and delivery is not well studied. Generally, it is recommended that blood pressure and heart rate be monitored routinely in all patients with CHD. Pulse oximetry may be important in patients with shunts or pulmonary hypertension; telemetry in those with history of arrhythmia, severe valvular lesions or ventricular dysfunction. Due to significant fluid shifts in the early postpartum period (described elsewhere in this review), monitoring should continue for 24–48 h postpartum. Leg compression devices and early ambulation are also important in this period to prevent venous thrombosis. As previously mentioned, use of micron-filter intravenous lines and meticulous intravenous care to prevent air or thrombotic emboli is important in the setting of shunt lesions [2]. 

Neuraxial anesthesia is often the preferred method of anesthesia in labor and delivery. Epidural anesthesia may cause systemic vasodilation, which may be dangerous in patients with severe aortic valve or subaortic stenosis. Slow induction and careful monitoring of blood pressure are believed to mitigate this risk. Similarly, when general anesthesia is necessary, careful induction may mitigate the negative inotropic effects of the anesthetic agents [63].

ACHD patients are at increased risk of postpartum hemorrhage, especially if they undergo cesarean delivery [6,62]. Ergometrine and carboprost, potent vasoconstrictors, may cause dangerous rises in pulmonary and systemic vascular resistance. It is recommended that they be avoided in patients with CHD [63]. Sulprostone and misoprostol are considered safe.

## 9. Conclusions

Accomplishing a successful pregnancy in the setting of CHD is possible in most cases with appropriate guidance and care. CHD is associated with increased risk of both maternal and fetal complications and mortality. However, with a carefully laid out plan, close follow-up and a multidisciplinary team approach involving CHD specialists, maternal fetal medicine, anesthesia and relevant subspecialties, outcomes may improve.

## Figures and Tables

**Table 1:** Predictors of adverse maternal cardiovascular events during pregnancy [2,11,12,13].

Modified WHO (Maternal Risk)	CARPREG II	ZAHARA
**Class I (2.5–5%):** Small or mild: pulmonary stenosis, patent ductus Arteriosus, mitral valve prolapse Successfully repaired simple lesions (atrial or ventricular septal defect, patent ductus arteriosus, anomalous pulmonary venous drainage), Atrial or ventricular ectopic beats, isolated **Class II (5.7–10.5%):** Unoperated atrial or ventricular septal defect Repaired tetralogy of Fallot; Most arrhythmias (supraventricular arrhythmias); Turner syndrome; without aortic dilatation **Class II–III (10–19%):** Mild left ventricular impairment (EF > 45%); Hypertrophic cardiomyopathy; Native or tissue valve disease not considered WHO I or IV (mild mitral stenosis, moderate aortic stenosis), Marfan or other HTAD syndrome without aortic dilatation Aorta < 45 mm in bicuspid; aortic valve pathology; Repaired coarctation Atrioventricular septal Defect **Class III (19–27%):** Moderate left ventricular impairment (EF 30–45%); Previous peripartum cardiomyopathy without any residual left ventricular impairment; Mechanical valve Systemic right ventricle with good or mildly decreased; ventricular function Fontan circulation. If otherwise the patient is well and the cardiac condition Uncomplicated; Unrepaired cyanotic heart Disease; Other complex heart disease; Moderate mitral stenosis Severe asymptomatic aortic Stenosis; Moderate aortic dilatation (40–45 mm in Marfan syndrome or other HTAD; 45–50 mm in bicuspid aortic valve, Turner syndrome ASI 20–25 mm/m^2^, tetralogy of Fallot < 50 mm); Ventricular tachycardia **Class IV (40–100%):** Pulmonary arterial hypertension; Severe systemic ventriculardysfunction (EF < 30% or NYHA class III–IV); Previous peripartum cardiomyopathywith any residual left ventricular impairment Severe mitral stenosis Severe symptomatic aortic stenosis Systemic right ventricle with moderate or severely decreased ventricular function; Severe aortic dilatation (>45 mm in Marfan syndrome or other HTAD, >50 mm in bicuspid aortic valve, Turner syndrome ASI > 25 mm/m^2^, tetralogy of Fallot > 50 mm); Vascular Ehlers–Danlos Severe (re)coarctation; Fontan with any complication	Prior CV events or arrhythmia (3 points) NYHA class > II or cyanosis (resting oxygen saturation < 90% at rest) (3 points) Mechanical valve (3 points) Systemic ventricular dysfunction with LVEF < 49% (2 points) High-risk left-sided obstruction (peak LVOT > 30 mmHg, mitral valve area < 2 cm^2^, aortic valve area < 1.5 cm^2^; (2 points) Pulmonary hypertension (RVSP > 49 mmHg) (2 points) Coronary artery disease (2 points) High-risk aortopathy (2 points) No prior cardiac intervention (1 point) Later pregnancy assessment (2 points) Score: 0–1 → 5% 2 → 10% 3 → 15% 4 → 22% ≥4 → 41%	history of arrhythmia (1.5 points) above II NYHA FC (0.75 points); LVOT obstruction with a peak > 50 mmHg or aortic valve area < 1 cm^2^ (2.5 points) mechanical valve prosthesis (4.25 points) moderate/severe systemic atrioventricular valve regurgitation moderate/severe sub-pulmonary atrioventricular valve regurgitation use of cardiac medications pre-pregnancy repaired or unrepaired cyanotic heart disease (1 point) Score: 0 to 0.5 points—2.9% 0.51 to 1.50 points—7.5%—1.51 to 2.50–17.5% 2.51 to 3.50–43.1% ≥3.51–70.0%

EF—ejection fraction; FC—functional capacity; HTAD—hereditary thoracic aortic Disease; LVOT—left ventricular outflow tract; NYHA—New York Heart Association; WHO—world health organization.

**Table 2:** Patients who should avoid Estrogen-containing contraceptives [4,16,19,20].

2018 AHA/ACC + 2020 ESC ACHD Guidelines	ACOG 2019 Practice Bulletin for Co- Existing Medical Conditions	US Medical Eligibility Criteria for Contraceptive Use, 2016
Prior thrombotic events Cyanosis Fontan physiology PAH Mechanical valves	Smoking and age 35 years or older Less than 21 days after giving birth 21–42 days after giving birth with peripartum cardiomyopathy History of DVT or PE Hereditary thrombophilia (eg anti- phospholipid syndrome) Superficial venous thrombosis (acute or history) Diabetes > 20 years or diabetes with microvascular disease	Multiple risk factors for atherosclerotic cardiovascular disease (e.g., older age, smoking, diabetes, hypertension, low HDL, high LDL, or high triglyceride levels) Hypertension Category 3: BP 140–159/90–99 Category 4: BP > 160/100 Vascular disease History of or acute DVT/PE Superficial venous thrombosis (acute or history) Ischemic heart disease Stroke Complicated valvular disease (PH, risk for afib, subacute bacterial endocarditis) Peripartum cardiomyopathy Severe liver cirrhosis

DVT—deep vein thrombosis; HDL—high density lipoprotein; LDL—low density lipoprotein; PE—pulmonary embolism; PAH—pulmonary arterial hypertension; PH—pulmonary hypertension.

**Table 3:** Congenital valvular disease in pregnancy [24,25,26,27,28,29,30].

Lesion	Maternal Complications	Fetal Complications	Considerations
Severe mitral stenosis	Mortality 3% HF 37% Arrhythmia 16%	Pregnancy loss 6% Preterm delivery 18%	Moderate mitral stenosis is mWHO risk class III; severe mitral stenosis is class IV (pregnancy contraindicated)
Severe aortic stenosis	Mortality 2% HF 9% Arrhythmia 4%	Pregnancy loss 5% Preterm delivery 4%	Severe symptomatic aortic stenosis is mWHO risk class IV (pregnancy contraindicated). Assisted second stage may be considered during delivery
Severe pulmonary stenosis	Thought to be well-tolerated based on limited data. Worsening functional status may occur	No significant effect observed in small study	
Moderate/severe atrioventricular valve regurgitation	Mortality <1% HF 8–11% Arrhythmia 6–8%	Pregnancy loss 0–1% Preterm delivery 12–15%	pulmonary hypertension or LV dysfunction portend worse prognosis
Moderate/severe semilunar valve regurgitation	Mortality <1% HF 1–3% Arrhythmia 0–3%	Pregnancy loss 1–8% Preterm delivery 5–10%	pulmonary hypertension or LV dysfunction portend worse prognosis
Bioprosthetic valve	Mortality 1% HF 7% Arrhythmia 5%	Pregnancy loss 2% Preterm delivery 12%	Left-sided prosthetic valve dysfunction portends worse prognosis
Mechanical prosthetic valve	Mortality 1% HF 8% Arrhythmia 3% Hemorrhage 23%	Pregnancy loss 18% Preterm delivery 18%	VKA use portends higher risk of adverse fetal outcomes; heparin use portends higher risk of adverse maternal outcomes
Endocarditis	Mortality 11%Septic embolization ~10–20%	Pregnancy loss 14%	Left-sided valve involvement portends worse prognosis; surgery performed in ~50%

HF—Heart Failure; VKA—vitamin K antagonists.

**Table 4:** Specific congenital heart disease lesions in pregnancy [2,43,44,45,46,47,48,49].

Lesion	Maternal Complications (Cardiac Event Includes MI, CVA, Death)	Fetal Complications	Considerations
Atrial septal defect	Cardiac event: 1.3% Arrhythmia/HF < 1% Endocarditis 3.8% TE 5% Pre-eclampsia 0.8%	Fetal mortality 2.4% Preterm delivery 6% CHD recurrence 6%	- DVT prophylaxis in all, AC consideration in high-risk patients for TE (e.g., atrial arrhythmia, thrombophilia, indwelling intra-cardiac devices). - Meticulous intravenous care. - Consideration of aspirin
Ventricular septal defect	Cardiac event: 1.2% Arrhythmia/HF < 1% TE 1.8% Pre-eclampsia 1.8%	Fetal mortality 1.4% Preterm delivery 11.8% CHD recurrence 2.7%	
Tetralogy of Fallot	Cardiac event: 0% HF 2.4% Arrhythmia 6.4% Endocarditis 0.6% TE—0.6% Pre-eclampsia 1.8%	Fetal mortality 0.5% Preterm delivery 6.3% CHD recurrence 3%	Elective PVR may be considered in large RVs and/or RV dysfunction.
Coarctation of the aorta	Cardiac event: <1% Pre-eclampsia 4.9% HTN—5.3–30% HF 1–3.3% Arrhythmia—0–1% endocarditis/TE—0% Aortic dissection (especially Turner syndrome)	Fetal mortality < 1% Preterm delivery 3–7.9% CHD recurrence 4%	Severe CoA is considered mWHO class IV (contraindicated). Control blood pressure during pregnancy without lower body perfusion compromise.
Ebstein Anomaly	Cardiac event: 0% HF 3.1% Arrhythmia 3.9% Endocarditis 0 % TE—0.6% Pre-eclampsia 1.8%	Fetal mortality 0% Preterm delivery 22% CHD recurrence 4%	Assess for cyanosis (PFO/ASD are common) and arrhythmia (WPW). Degree of TR and RV function determine outcomes.
d-transposition of the great arteries	Data primarily pertain to atrial switch (i.e., Mustard, Senning). Arterial switch data (ASO) are emerging as reassuring. Cardiac event: 2.2% HF 9–10.8% Arrhythmia 15.6% Endocarditis 0% TE—3% Pre-eclampsia 10.3%	Fetal mortality 2.8% Preterm delivery 34–38% CHD recurrence 0.6%	- Confirm no ischemia in ASO prior to pregnancy - In atrial switch, Degree of TR, systemic ventricle function determine prognosis.
Congenitally corrected transposition of the great arteries (cc-TGA/L-TGA)	Cardiac event: 2.4% HF 7.1% Arrhythmia 3.6% Endocarditis 1.2% TE—3.7% Pre-eclampsia 2%	Fetal mortality 1.3% Preterm delivery 9% CHD recurrence 3.6%	Assess the systemic RV function, degree of tricuspid regurgitation, WPW and potential of heart block
Cyanotic conditions	Cardiac event: 4% HF 18.9% Arrhythmia 4.8% Endocarditis 4.1% TE—3.6% Pre-eclampsia 0%	Fetal mortality 12.2% Preterm delivery 44.6% CHD recurrence 7.4%	Contraindicated for pregnancy. Care includes filtered vascular lines, compression stockings, sequential compression devices, treatment of iron deficiency and thromboembolic prophylaxis.
Eisenmenger syndrome	Cardiac event: 33% HF 21.1% Arrhythmia 0% Endocarditis 0% TE—18.8% Pre-eclampsia 0%	Fetal mortality 9.9–30% Preterm delivery 65% CHD recurrence 5%	- Contraindicated for pregnancy. - endothelin receptor antagonists and riociguat are contraindicated for use in pregnancy - PDE-5i and prostanoids may be used as advanced PH medications. - see care of cyanotic conditions
Single ventricle palliated with a Fontan circulation	Cardiac event: <1% HF 3–11% Arrhythmia 3–37% Endocarditis 0% TE—0% Pre-eclampsia 0%	Fetal mortality 0–5% Preterm delivery 28–59% Post partum hemorrhage 14% CHD recurrence 4%	- Fontan with complications is contraindicated to proceed with pregnancy - Anticoagulation is recommended to all pregnant Fontan patients.

AC—anticoagulation; ASO—arterial switch operation; CHD—congenital heart disease; CVA—cerebrovascular accident; DVT—deep vein thrombosis; HF—heart failure; MI-myocardial infarction; PDE-5i—phosphodiesterase-5 inhibitor; TE—thromboembolism; TR—tricuspid regurgitation.

## Data Availability

Not applicable.
